# Limitations of Using Mobile Phones for Managing Type 1 Diabetes (T1D) Among Youth in Low and Middle-Income Countries: Implications for mHealth

**DOI:** 10.1145/3687045

**Published:** 2024-11-08

**Authors:** SUSAN WYCHE, JENNIFER OLSON, MARY KARANU, ERIC OMONDI, MIKE OLONYO

**Affiliations:** Michigan State University, Department of Media and Information, USA; Michigan State University, Department of Media and Information, USA; Rural Outreach Africa, Kenya; Kenya Diabetes Management & Information Centre (DMI); County Government of Vihiga, Department of Health, Kenya

**Keywords:** Design, Diabetes, Kenya, mHealth, mobile phones, user studies

## Abstract

The prevalence of Type 1 Diabetes (T1D) among youth is increasing worldwide. Mobile phones, particularly mHealth applications, can potentially improve youth’s management of this chronic condition. However, the design of these services rarely accounts for users in low and middle-income countries (LMICs). In this paper, we investigate factors that influence the use of mobile phones for managing T1D among youth in rural and urban Kenya. Our analysis draws from 58 interviews conducted with T1D youth (between the ages of 11 and 18 years old), their caregivers, and other significant stakeholders, including doctors and schoolteachers. Our findings draw attention to a significant mismatch between the mobile phone features prioritized in mHealth apps and participants’ usage practices. We discuss the practical implications of these findings for mHealth design and user research.

## 1 INTRODUCTION

The revolutionary growth in mobile phone ownership has spurred significant interest in developing mobile health, or mHealth applications (henceforth referred to as “apps”) to address the global challenge of type 1 diabetes (T1D). T1D is a non-communicable and chronic condition that impacts 8.4 million people worldwide; this number is projected to double by 2040 [26]. The condition is typically diagnosed in childhood and demands continuous self-management throughout one’s life. This management includes monitoring blood sugar levels and injecting insulin multiple times a day, adhering to a balanced diet and exercise routine, and meeting regularly with a healthcare provider.

Poor management can lead to serious health complications, a diminished quality of life and a high risk of mortality. Mobile phones and apps are widely recognized as powerful tools that can improve T1D self-management [47]. This is especially true for adolescents, who extensively use these devices [11] and who encounter particular challenges managing T1D at this developmental stage [10].

The fields of HCI and CSCW have maintained a longstanding interest in diabetes. Researchers have contributed to the development of mHealth technologies [1, 9, 12, 14], and have conducted user studies to explore how people’s management of T1D can inform the design of these technologies [38]. To date, this research has primarily been conducted in high-income countries in Europe or North America, with few studies investigating mHealth and T1D management in low and middle-income countries (LMICs). The purpose of this study is to explore the factors influencing mobile phone use among youth with T1D, and their caregivers, in a LMIC—specifically, Kenya in East Africa. Similar to other LMICs, T1D prevalence among adolescents is rising and mobile phones are widely accessible in Kenya [47, 54]. Understanding the factors affecting Kenyan youth and their caregivers’ interactions with mobile phones is essential for designing effective interventions that can support T1D management. Such studies are also important for understanding if mHealth interventions can actually improve health outcomes for this population and those in comparable contexts.

In this study, we interviewed 23 early to middle adolescents (henceforth “youth”), between 11- and 18-years old (y.o.), their caregivers (n=25), as well as 10 key stakeholders who interacted with these participants (e.g., doctors, community health workers, nurses, and schoolteachers). We collected data in Nairobi, Kenya’s capital city, and in Vihiga County, a rural area in Western Kenya. Our findings draw attention to limited use of mobile phones to support T1D. This limited use was primarily due to a significant mismatch between the mobile phone features prioritized in mHealth app design [38], and participants’ usage practices. Our findings also demonstrate how mobile phones support T1D care in ways not anticipated in the HCI/CSCW communities; in particular, participants’ preference for voice calls to short message service or SMS. We discuss the practical implications of our findings. These include, encouraging HCI/CSCW researchers to reevaluate the potential of mHealth and to recognize the implications of developing mHealth apps that are based on design principles which are unlikely to apply to “majority world” users in Kenya and other LMICs (e.g., deepening, rather than disrupting, global health disparities). Lastly, we discuss this study’s limitations and make future research recommendations.

This paper contributes to HCI/CSCW by providing new understandings of mobile phone use and T1D management in Kenya; in particular, how these topics relate to the use of mHealth apps. More broadly, this research deepens these communities’ global understanding of these topics, and their implications for design and research.

## 2 RELATED WORK

### 2.1 Mobile Health (mHealth)

Reports indicate that 73% of the world’s population, over 10 years old, own a mobile phone [47]. This growth has motivated significant interest in mobile health, or mHealth—a term that broadly refers to using mobile devices for health-related purposes; the field encompasses using SMS, voice calls, wearable computers, and—especially—mobile apps to provide people with health information [34]. According to Klasnja and Pratt, mobile phones have unique features that make them powerful tools for improving health outcomes [38]. These scholars argue that the “personal nature” of phones; that is, people’s tendency to always have their own devices with them increases users’ acceptance of mHealth interventions. This accessibility allows users to readily access health information via the internet. Indeed, this “always-on connectivity” is regarded as especially useful when developing apps to support T1D management, because users can immediately upload their blood sugar level readings to a healthcare provider’s server, so that critical events (e.g., hyperglycemia and hypoglycemia^1^) can be quickly detected.

SMS is another feature that has played a significant role in the development of mHealth services, especially in LMICs. Klasnja and Pratt write “SMS is widely used not only in the first world but in developing countries” [38]. They add that this feature is efficient and “universally accessible” because it works on basic, feature and/or smartphone models. It is also generally perceived as low-cost when compared to other information delivery methods (e.g., voice calls) [47] and can be used to “push” health information to users [24, 34]. This knowledge has motivated multiple efforts to develop and evaluate SMS-systems in Kenya, and other LMICs, that send patients reminder messages (e.g., to take medications and attend medical appointments) [6, 28, 29, 53, 73], and health education information (e.g., eat healthy and stay hydrated) [1, 17, 41]. Reviews of these studies suggest that these services primarily focus on providing information to people with communicable diseases (e.g., HIV/AIDs) and that more evidence is needed to assess the impacts these systems have on health outcomes [16, 27, 30]. Our research builds upon these studies by examining T1D—a non-communicable condition. We also investigate mobile phone use more broadly, to understand its impacts on mHealth apps.

#### 2.1.2 T1D Apps and Adolescents

Diabetes is often noted as the most popular chronic clinical condition targeted by mHealth. There are an estimated +1000 diabetes apps on the Google Play store (e.g., Glucose Buddy, mySugr, and On-Track Diabetes) [14, 21]. This number is expected to grow [33]. Scholars have reviewed these apps [4, 12, 15, 20], and found that these services typically include features which support users logging/charting their blood sugar readings, insulin dosages, and foods eaten. Other common features include providing users with educational tips and advice on how to manage T1D [22, 36, 45]. A growing number of these apps integrate social media into them. Facebook, Twitter^2^, Instagram, and other platforms can offer patients opportunities to learn about their condition and to communicate with others with it [62, 63]. It is also becoming more common for these apps to support data upload/synchronization with glucometers or insulin pumps (e.g., Glooko) [19]. These reviews overwhelmingly focus on apps developed for users in the U.S. and other high-income countries [3, 74]; they exclude apps designed for people elsewhere, including in Kenya where services have been developed for people with T1D (i.e., Afya Pap).

These reviews also suggest that adolescents and young adults with T1D are generally the targets of mHealth apps. This demographic, between the ages of 10 and 19 y.o., is regarded as owning and frequently, if not excessively, using mobile phones to pass time, connect with others, and to learn new things, including health information [75]. Further, this developmental period, which broadly encompasses youths’ transition to adulthood, is marked by physical, emotional, and social changes that complicate effective T1D management (e.g., peer pressure, assertion of independence, and competing demands) [10]. mHealth apps are presented as desirable solutions that are both readily accessible and socially acceptable [38]. However, questions persist about their impact on health outcomes, and it is suggested that—at best—their results are mixed [23, 72]. Our study contributes further evidence regarding the potential of mHealth to improve health outcomes among adolescents with T1D in Kenya.

### 2.2 HCI/CSCW and Diabetes

HCI and CSCW researchers have long recognized mHealth’s potential in improving health outcomes for people with T1D; many of the aforementioned features have guided the development of mobile systems for users with the condition [43, 56, 57]. More recent design efforts include developing systems that rely on more sophisticated technologies to support T1D management, these include using artificial intelligence (AI) to predict blood sugar levels [35], employing machine learning (ML) to provide users with personalized management suggestions [35, 69] and to design models that respond to diabetics’ care needs [4].

The enthusiasm for mHealth’s potential in supporting T1D management has also generated significant interest in conducting user studies examining how people manage the condition. Early user studies include Mamykina et al.’s investigation of adults’ “judgments and reasoning” in diabetes self-management. Their findings motivated various design recommendations, including intelligent visualizations which communicate the relationship between patients’ daily activities and blood sugar levels [44]. As interest in mHealth and diabetes management has grown, so too has the HCI/CSWC communities’ interest in studying how particular user groups, including adolescents and their caregivers, manage the condition. Recognizing the significant role that caregivers play in supporting young children with the T1D, Cha et al. interviewed these users. They identified parents’ strategies for helping their children maintain healthy blood sugar levels and encouraged the development of self-tracking tools which monitor children’s abilities to self-manage T1D [12, 13]. Raj et al. also studied caregivers of adolescents with T1D. They found that context (e.g., school, home, work, as well as time of day/month/year) influenced participants’ self-management behaviors [65]. These studies provide important insights into how to design mHealth and related technologies for people with T1D. However, their focus on the “minority world”; that is, primarily high-income countries tell us little about how most of the world uses mobile phones in relation to T1D. Indeed, the lack of research—especially qualitative studies—investigating T1D in LMICs; in particular, in African countries has been observed in other fields (e.g., public health) [52, 58].

In response to this limitation, there have been some efforts within HCI and CSCW to investigate T1D and technology use in LMICs, as well as countries outside of Europe and North America. Notable examples include Hentschel et al.’s study of middle-income diabetes-affected households in India [32]. Other examples include Chen’s cross-cultural investigation of how diabetics use health information to manage their condition; her study included field sites in the U.S. and in China [15]. Indeed, China has the world’s largest diabetic population, a factor which motivated Zhou et al. to study *Sweet Home* a popular online health community in the country. Their findings detail how attitudes towards traditional Chinese medicine, cultural norms about eating, and other factors influence diabetes care. They conclude that there is a need for researchers to consider diabetes in a broad range of “social and cultural settings” [85]. Stowell et al. reviewed prior research examining the design and evaluation of mHealth technologies for vulnerable populations. Their conclusions included a call for HCI/CSCW researchers to examine mHealth “from a global perspective,” adding that such research can provide “valuable directions” for the fields [70]. Similar to these prior studies we use qualitative methods to explore how people manage diabetes in their everyday lives and reflect on their implications for design. We also respond to calls to study diabetes in different settings by studying youth with T1D and their caregivers in Kenya.

## 3 METHODS

This study is part of an ongoing project aimed at designing an intervention that supports Kenyan adolescents’ T1D management. Formative research began in April 2022. Our goal was to explore youth and their caregivers’ experiences with T1D; in particular, how they used (or did not use) mobile phones to manage the condition. We conducted semi-structured interviews, because this method provided a flexible approach which allowed for in-depth exploration of diverse experiences related to T1D and technology use [9]. Our data include 23 semi-structured interviews with youth (14 boys and 9 girls), 25 with their caregivers (23 women and 2 men), and 10 key stakeholders (e.g., doctors, nurses, and schoolteachers) (5 women and 5 men).

### 3.1 Study Setting

Kenya is classified as a middle-income country in East Africa; however, 18 percent of the country’s population lives below $1.90 U.S. per day, most of whom are in rural areas [64]. Similar to other African countries, T1D diagnoses are rising in Kenya, especially among middle-to-late adolescents (13–18 y.o.). An estimated 50,000 to 70,000 Kenyan youth have T1D, and this number is rising [25]. Accurate numbers, however, are difficult to obtain and it is likely that many youth with the condition have not been diagnosed, due to the country’s poorly equipped health-care system [60]. The authors’ familiarity with, and extensive experience living in and conducting research in the country, were the primary reasons we conducted this study in Kenya. The country is also home to our project partner. Kenya Diabetes Management & Information Centre, also known as “DMI”, is a Nairobi-based NGO which administers screening tests for diabetes, educates health care providers about the condition, and conducts diabetes research in East Africa.

This study draws upon 58 in-person interviews conducted in two distinct locations: Vihiga County (n=28), a lower-income agricultural region in western Kenya, and Nairobi (n=30), the country’s capital and largest city. Rural areas, like Vihiga County, typically have fewer job opportunities, lower population densities, and limited infrastructure. Conversely, urban areas like Nairobi generally offer better access to healthcare facilities and medical professionals. Collecting data at a rural and an urban site was useful for gaining some understanding of how these differences affect T1D management.

### 3.2 Participant Recruitment and Data Collection

DMI maintains a database with ~400 youth with T1D. To ensure diverse perspectives, we aimed to recruit equal numbers of girls and boys, as well as participants from different socio-economic backgrounds (class, religion, and ethnic group). DMI used their database to identify 23 adolescents (between 11 and 18 y.o.), who had been diagnosed and living with T1D diagnosis for at least one year, lived in one of our field sites, and who were able to participate in this interview and other phases of the study. Roughly half of the 23 youth were from Nairobi (n=12) and 14 were boys (9 girls); 11 youth were from Vihiga (6 boys and 5 girls). We also invited participants’ caregivers to participate, because they tend to play a significant role in youths’ management of T1D (n=25) [71]. Similar to the youth, roughly half of these participants lived in Nairobi (n=13), however, most of these individuals were women (n=23). Other stakeholders were interviewed because they interact with youth during clinical care and when they are at school. Two of the doctors and two of the community health workers interviewed had experience caring for youth with T1D in both rural and urban areas; we interviewed one teacher and school nurse in Nairobi and two teachers and two school nurses in Vihiga.

Caregivers, youth, and stakeholder interviews were conducted separately; however, we used a similar interview protocol during all interviews. We began by collecting demographic data (age, occupation, grade level, etc.) We then asked youth and caregivers questions about when they learned about their diagnosis, how they managed the condition (e.g., how frequently they tested their blood sugar levels and injected insulin), and how having T1D impacted them and their families’ everyday lives (e.g., diet and relationships). We asked youth questions about their experiences in school. All interviews included questions about accessing information and mobile phone use; in particular, what type of phones participants had and what they used it for (accessing social media, searching online, etc.). We asked stakeholders questions about their experiences engaging with T1D youth, typically at schools or health clinics. Considering that our research was conducted in the aftermath of COVID-19, we asked participants how school closures, lockdowns, and other events related to the pandemic affected T1D management.

All interviews were conducted by trained interviewers paired with experienced translators. Participants were bilingual or multilingual language speakers. They were encouraged to speak in the language they felt most comfortable with, resulting in most interviews (n=44) being conducted in Kiswahili, with instances of code-switching (alternating between two languages) in all sessions. The interviews typically lasted 30 to 45 minutes.

### 3.3 Data Analysis

Interviews were digitally recorded, transcribed verbatim and translated into English—if necessary—by experienced Kenyan transcribers. An inductive and iterative process was used to analyze these transcripts [11]. The first and second authors listened to the interviews and read through the transcripts two to three times to gain familiarity with the data and to identify initial coding ideas. We then created codes that were based on specific words and phrases from the interviews (e.g., phones at school, misinformation, and technology non-use), and wrote memos that connected these emerging observations with these codes. After this, we made comparisons between interviews and codes representing similar ideas, and then grouped these into thematic categories. We then took quotes from the interviews and placed them in the appropriate category to illustrate the themes, and to ensure that they were grounded in the data. Comparing our findings with those reported in prior research strengthened our analysis. Emerging themes were further clarified through multiple phone calls and/or face-to-face meetings with co-authors, youth, their caregivers, and doctors. We then developed the final categories presented here.

### 3.4 Ethics Approvals and Positionality

The study received ethics approvals from the African Medical and Research Foundation (AMREF), the Kenya National Commission for Science and Technology, and Innovation (NACOSTI), and Michigan State University’s institutional review board (IRB). Permissions were also sought from government health officials in our field sites. Researchers provided and read aloud consent forms to prospective participants, before interviews. Parents and/or guardians signed these forms and youth gave verbal assent. Interviewees were assured that participation in the research was voluntary and that they could withdraw at any time. No financial incentives were offered; however, upon completion of the interview youth received a digital camera (valued at $35)^3^.

Researcher self-disclosure is typically regarded as ethically sound and valuable for improving the validity of qualitative research [66]. Here, and throughout the paper, we share aspects of our positionality; that is, information about the researchers’ backgrounds and experiences. The first and second authors have been educated and socialized in the U.S. They have a combined 45+ years experience living and conducting research in Kenya and other African countries. Their familiarity with the context was useful for identifying collaborators, designing the study, collecting data, and accurately interpreting it. The third author is Kenyan; she was educated in the U.S., is fluent in English and Kiswahili, and has extensive experience conducting interviews. The fourth author is also Kenyan; he has lived with T1D for over 20+ years, is a diabetes educator and respected expert on the condition. The fifth author is a medical doctor based in rural Kenya; he routinely treats youth with T1D. Our research team maintained a collaborative approach throughout the project. We jointly developed the interview protocol, recruited participants, conducted interviews, and analyzed data.

## 4 FINDINGS

The findings are organized as follows. We begin by providing an overview of participants’ mobile phone ownership and what kind of devices they had (e.g., smart or basic phone). Next, we detail why most of the youth and their caregivers did *not* use mHealth apps; in fact, few used mobile phones to manage and/or to learn about T1D due to infrastructural and socioeconomic factors, such as bans on devices in Kenyan schools. Then, we describe how the youth in our study predominantly used their phones: that is, for leisure activities (e.g., playing games and watching videos). Participants rarely searched online for information about how to manage their T1D. One possible reason for this could be their deep understanding of the condition. This knowledge primarily came from managing the condition for at least a year, as well as their regular interactions with trusted doctors and healthcare providers. Instead, it was participants’ schoolteachers who most needed information about T1D. Then, we explain the most common ways phones were used in relation to T1D: that is, for making and receiving voice calls. These findings draw attention to significant discrepancies between the mobile phone features emphasized in mHealth applications and interviewees’ actual mobile phone usage practices.

### 4.1 Owning and Using Mobile Phones

mHealth efforts are predicated on owning a mobile phone and being able to consistently use it to access the internet [38]. Of our participants, nearly every caregiver, half of the youth, and all stakeholders had a mobile phone. However, owning a phone did not necessarily mean that participants could use it to manage, or even to learn about T1D. To be useable a phone needs to be in working condition and have a charged battery. Accessing mHealth apps also requires owning a smartphone and having sufficient data bundles^4^ for internet access, both of which were uncommon, especially among rural participants. Shared devices and mobile phone bans at schools also limited using mobile phones for health-related purposes.

Forty-five of the 58 participants had mobile phones; however, we observed disparities in smartphone ownership between rural and urban participants. Ten of the 12 Nairobi youth reported having a mobile device; seven of these participants owned smartphones (typically Android models). Their caregivers also had mobile phones. All 13 reported having a device; 10 had smartphones. In Kenya, a low-quality smartphone can be purchased for $20-$27; yet, these devices remain prohibitively expensive, especially for participants living in rural areas. Four caregivers and one youth living in Vihiga County said they had a smartphone. Instead, it was more typical for these participants to own inexpensive basic/feature phone models, popularly known as *kabambe*. Eight rural caregivers and four youth had one of these devices. All key stakeholders owned smartphones.

Regardless of the phone model they had, few participants used their devices for health-related purposes. Factors that hindered phone and mobile internet use were identical to those reported in prior studies conducted in Africa. These factors included phones being broken, or “spoiled”; this was especially common in rural areas where cheap and second-hand phones are common [77, 82]. Maintaining a charged battery was also a challenge for some interviewees, because they did not have access to grid electricity; this was also more typical among participants living in rural areas [82]. Phone sharing was also common, especially among the youth. This practice was likely motivated by altruism, cultural norms, as well as lack of resources [39]. The youth in our study tended to have limited money, which made it difficult to buy their own phone. Nearly half of the youth interviewed told us that when they did use a smartphone, it was their caregivers’ device.

Not having a personal device likely hindered youths’ adoption of mHealth apps. Of course, participants with basic/feature phones were not using mHealth or other apps because these models do not support downloading and installing them. Even among those who had smartphones, just one participant had an mHealth app on his device. He told us that he rarely used the app because it required having sufficient airtime/credit on his smartphone. He explained:

If you can get an app without bundles, then you can do your thing, and upload your data, but you must have bundles to feed the app. There would be an advantage of having an app that does not need bundles.

(17 y.o. boy, Nairobi)

This participant described constantly having to “feed” his phone—by buying data bundles—as challenging. His comments also draw attention to another challenge: for most T1D apps to improve health outcomes, users must upload blood sugar level readings and other data multiple times a day [7]. Underlying the vast majority of these apps are assumptions about “always-on connectivity” which mostly did not exist for participants; instead, access was intermittent, and primarily based on when—and if—participants had airtime on their devices and/or a charged handset battery [83]. In turn, this meant that they were not engaging in other activities that are increasingly integrated into T1D mHealth apps, such as sharing their data with healthcare providers, or interacting with other youth on social media. Just one participant mentioned using Facebook to interact with other T1D youth. More broadly, it was typical for the youth and their caregivers to *not* interact with other diabetic youth, a finding which was likely exacerbated by the COVID-19 lockdowns. Participants did not use apps or the internet to share medical information with their healthcare providers, nor did they use mobile services that connect glucometer data with their mobile phones. In fact, just half of the interviewees had a functional glucometer they could use for self-testing. Similar to smart phones, these devices were unaffordably expensive. Further, owning a glucometer did not mean it was usable; often, the devices were broken, or lacked batteries and/or paper testing strips. Prior research carefully details these and other challenges affecting glucometer use in LMICs (see [40]).

Other circumstances hindered phone use. Notably, youth with phones could not regularly use them, because the devices are banned in Kenyan schools, as is common in other African countries [84]. Teachers explained the reasons for these bans, including that mobile phones can distract students from learning and that access to them is inequitable (e.g., not all students can afford a phone). This finding is noteworthy because attending school is a significant part youth’s lives. In Kenya, adolescents spend 8–10 hours a day, nine months a year at school. Many Kenyans, including most in our study, attend boarding schools, which are frequently far from their homes. Youth and their caregivers expressed frustration about these bans, and consistently shared stories of being unable to communicate with each other and/or their doctors when they ran out of insulin or were experiencing a medical emergency. As with the aforementioned socioeconomic and infrastructural factors which hinder phone use, prior HCI/CSCW studies have not accounted for this ban.

### 4.2 Useful Information and Who Needs It

Similar to prior studies of mobile phone use in Africa [49], we observed that youth rarely used mobile phones for “serious purposes” [67], nor did they use them to access so-called “useful information” which mHealth apps are supposed to provide [2]. One possible explanation for this observation was that interviewees were deeply knowledgeable of T1D and how to manage it. Indeed, all youth had been living with T1D for at least one year, and more than half (n=13) had been managing their condition for four or more years, including one 16 y.o. who had been living with T1D since he was six years old. Their knowledge primarily came from regular interactions (every three months) with their doctors and healthcare providers, rather than the mobile internet. When we asked youth about their phone and internet use, they primarily talked about gaming, entertainment, and personal development activities.

Girls described watching videos on *YouTube*, for example, a 15 y.o. who aspired to be a chef told us she viewed videos of people baking cakes. Some boys also said they watched cartoons; they also mentioned playing free mobile games. Other youth talked about searching for information about sports, typically football. This participants’ explanation of his phone use was representative of others:

For my phone it is, even when I just open my Google account here, you will see football, many, many, many of the times it is football.

(14 y.o. boy, Nairobi)

HCI scholars have found that in other LMICs, play serves as a powerful motivation for phone use. These researchers suggest that these usage practices are primarily driven by a desire to have fun [18]; this also likely explains our participants’ motivations for playing games, watching videos, etc. As previously mentioned, we found that youth and their caregivers generally did not rely on mHealth or the mobile internet to get health-related information because they knew how to effectively manage their T1D. However, they typically gained this knowledge *after* being diagnosed.

Prior to being diagnosed with T1D, both youth and their caregivers had limited—to no—knowledge about the condition. When recounting their initial diagnosis, almost all interviewees remember what precipitated them learning that they had T1D; this typically included experiencing severe complications (e.g., ketoacidosis) and rushing to a hospital. Nearly every youth and their caregivers remembered the exact date of their diagnosis and consistently used the term “nilishtuswa” (Kiswahili for being shocked or startled) to describe how they felt. This surprise was due to misconceptions about T1D; in particular, that it was a condition that only affected people living in high-income countries, older adults, or—less frequently—which was caused by witchcraft. Over time, and after gaining sufficient knowledge, youth and their caregivers generally accepted that while the condition was a challenging one, they could lead a “normal life” if they ate properly, exercised, monitored their blood sugar levels and regularly injected insulin. Indeed, youth knew which foods to avoid and which to eat, as well as the importance of physical activity, proper foot care, medication adherence, and regularly seeing their doctor or healthcare provider (e.g., every three months). Most participants were also able to identify symptoms associated with impending complications (i.e., hyperglycemia and hypoglycemia), and dismissed misinformation about T1D, for example:

There’s this one I heard recently that someone conducted research and found a cure for diabetes. Someone sent it to me and said that I should make an order, about 4,000 shillings per bottle. So, I gave out my number and I received a call. The information I received, and I had read were two different things, so I rejected. It was a scam. I told him no.

(35 y.o. woman, Vihiga County)

During interviews, we asked youth and their caregivers if they regularly searched online for information about T1D; the vast majority did not. Three youth recalled using Google to search for information about nutritional value of specific foods. Roughly a third of the participants mentioned having used the mobile internet to learn whether a cure existed for T1D (one does not). We probed to understand why participants rarely searched for information online; this mother’s response was similar to others:

I was searching on Google, and saw everything written in medical terminology, and it was in English. So, you know, sometimes this language is incomprehensible.

(42 y.o. woman, Vihiga County)

Prior research suggests that patients prefer information in their own language [42]; as our observations suggest, it is challenging to find online content about T1D that has been translated into Kiswahili. Although the youth and their caregivers generally did not search online for health information, this finding does not imply there was no need to educate people about T1D. mHealth apps are typically designed to deliver personal health information directly to patients: that is, individuals *with* T1D [38]. However, it was members of participants’ communities, especially schoolteachers, who urgently needed this information. This lack of knowledge in schools contributed to misunderstandings and stigma about diabetes; it also led to some youths’ social exclusion, and discrimination at school, as this mother explains:

The teachers know that she has a problem. Some think that this disease is infectious, so they don’t want to associate with her. And now, I feel now what can I do about that? The teachers don’t want to understand, the teacher is a problem, other children are also a problem. Where she keeps her medicine, sometimes she finds someone has misplaced the medicine. So, there are many challenges, especially in school. They don’t want to understand that she is diabetic.

(35 y.o. woman, Vihiga County)

In addition to not providing students suitable places to properly store their “medicine,” or insulin (i.e., a refrigerator), interviewees said that misunderstandings about T1D also made maintaining an appropriate diet difficult, and negatively affected their learning conditions. The teachers and nurses we interviewed generally confirmed these findings, telling us their limited knowledge of T1D was a challenge. Similar to the aforementioned findings, these observations draw attention to how assumptions underlying mHealth (e.g., that people with the condition are those most in need of health information) cannot be taken for granted in Kenya.

### 4.3 Voice Calls: Mobile Phones’ Most Important Feature

Participants most frequently used their mobile phones’ voice calling feature for managing T1D. Caregivers consistently emphasized the importance of being able to initiate and receive voice calls, especially during T1D-related emergencies. They also consistently explained why they preferred making voice calls to sending an SMS.

Caregivers detailed how they used their phone’s voice feature to support T1D care. They recounted instances of calling clinics and pharmacies to ask about the availability of insulin and other supplies essential for T1D management (syringes, cotton wool, alcohol, etc.). This was especially crucial in rural areas like Vihiga County where these supplies were not always available, due to inadequate funding for healthcare infrastructure and unreliable transportation networks [51]. The COVID-19 pandemic further aggravated supply shortages. As an illustrative example:

Corona, yeah it affected us, because medication was hard to get, moving from there to here, it was a problem. So, I called him because there was a shortage of insulin. That is when I asked him, if I can get some for the day but there was no insulin in this hospital, so I didn’t get it and I just went direct to the next town to get it.

(35 y.o. woman, Vihiga County)

Initiating a voice call and being able to immediately speak to someone meant that caregivers were confident they could get what they needed, and not waste time and money traveling long distances to find that supplies were unavailable. A more significant reason caregivers made voice calls was to get help during an emergency; in particular, when their child had “hypo” (or hypoglycemia). This medical condition occurs when blood sugar levels drop below a healthy range. Symptoms include sweating, dizziness, and fatigue. If not immediately treated it can cause a patient to collapse. Several factors can exacerbate hypo, including food insecurity and the stress of unstable housing [5, 48]. These emergencies tended to prompt caregivers to call a trusted doctor or healthcare provider, as illustrated by these responses:

To communicate with the doctor when I have a problem, or emergency, I call the hospital and report the child is in such and such situation. Then I tell them he’s in a serious condition, and I ask them what to do, and they always advise me to take him to the hospital quickly, and I rush him there. The phone is very useful. I can seek help from people through the phone.

(43 y.o. woman, Vihiga County)

Maybe the sugars are low, what to do, when the child is sick. Yeah, I can seek help from other people in terms of communicating through the phone.

(35 y.o. woman, Vihiga County)

In addition to using their phones to “seek help” during an emergency, caregivers also received voice calls when their child experienced a crisis. These calls typically came from schoolteachers and/or administrators, as these parents describe:

When they see her getting sickly or weak at school, they would call me immediately and I pick her, then I would take her to hospital.

(32 y.o. woman, Vihiga County)

The teachers sometimes call me from school to say come and see your daughter, she has collapsed.

(41 y.o. man, Nairobi)

Given the limited access to emergency healthcare in Kenya, especially in rural areas, it becomes clear how important voice calls are for seeking immediate advice from trusted healthcare providers. The doctors and healthcare workers interviewed also recognized that voice calls were caregivers’ preferred way to communicate. They added that they received between five and seven calls during a typical day and sometimes at night, with the most common calls being about hypo-related emergencies. These participants explained that they routinely provided callers with step-by-step instructions on what to do if a youth was experiencing symptoms associated with dangerously low blood sugar levels (e.g., stop all activities, measure blood sugar levels, eat something, and in some cases immediately go to a hospital).

These stakeholders had smartphones and frequently used *WhatsApp* for personal and professional purposes. However, all recognized that this popular instant messaging service was an ineffective platform to use when communicating with some patients and their caregivers. Significantly, *WhatsApp* was not useful for communicating with people who had kabambe. Participants consistently discussed other reasons that they preferred voice over SMS communication. Caregivers told us that they often ignore SMS messages, because they frequently come from unfamiliar senders, or are spam messages (e.g., Safaricom advertisements). Further, and as observed in prior research, sending an SMS “takes time” [78–81]. The process of inputting text is slow and cumbersome, making it impractical for delivering urgently needed information.

Finally, prior research suggests that coordinating between patients and clinicians makes voice calls challenging [38]; however, this was not the case among our participants. The doctors and healthcare workers readily provided their contact information to youth with T1D and their caregivers and encouraged patients to call them during emergencies. They also perceived being available to answer calls as an inherent part of their job—a finding reported in other studies of healthcare workers in Africa [31]. Similar to other findings, these also suggest that the prioritization of certain design features in mHealth, such as a preference for SMS due to its efficiency in communication, may not align with participants’ practices.

## 5 DISCUSSION

This study of the factors affecting mobile phone use among Kenyan youth with T1D, and their caregivers, has broader implications for mHealth design and research. Our findings provide compelling evidence that there is a significant mismatch between the mobile phone features prioritized in mHealth apps and participants’ usage practices. In particular, mobile phone ownership may be widespread, but it is not universal. Statistics on phone ownership are often used to motivate the development of mHealth apps for users in LMICs. However, they provide an incomplete picture that fails to consider broken phones, disparities in phone ownership between rural and urban areas, mobile phone bans in schools, shared devices, and the financial challenges associated with buying data bundles. More broadly, youth and their caregivers did not seem to need mHealth apps, nor did they rely on the mobile internet to learn about managing their condition. Participants said that online diabetes-related medical information in their local languages was limited to nonexistent. Further, they all generally knew how to manage their T1D and had access to trusted healthcare providers. Instead, the people who most needed information about T1D were those who youth spent the most time around, particularly schoolteachers. Our finding support Bhat and Kumar’s observation, that cultural and ecological factors—in LMICs—are sometimes ignored in digital health applications [8].

We observed some benefits of mobile phones, especially being able to call doctors during T1D-emergencies, but this aspect of mobile phone use has been downplayed in discussions about mHealth, in favor of what are *perceived* as more accessible and efficient forms of communication (i.e., SMS). The discovery that caregivers prefer voice calls over SMS contradicts findings from prior HCI/CSCW studies which suggest that SMS is the most effective means of delivering health information in LMICs [20, 55, 61]. Our novel findings about *WhatsApp* usage draw attention to the ways this popular platform complicates knowledge about SMS and universal accessibility (i.e., *WhatsApp* messaging is incompatible with basic and feature phones). Finally, our research provides some insights into how the global COVID-19 pandemic affected T1D care in Kenya.

Stowell et al. have urged researchers to examine mHealth “from a global perspective,” because doing so can provide “valuable directions” for the HCI/CSCW fields [70]. Our findings draw attention to these directions: encouraging researchers to reevaluate the potential of mHealth and to recognize how developing mHealth apps—which are based on design principles that are unlikely to apply to majority world users worldwide—may deepen, rather than disrupt, global health disparities.

### 5.1 Implications for mHealth Design: Reconsidering Apps

These findings contribute to an emerging discourse that raises questions about the effectiveness of mHealth, especially for T1D care, and in Africa. These studies conclude that mHealth apps are generally not designed to meet users’ needs [37] and that there is insufficient evidence to demonstrate that they improve diabetes health outcomes [36, 72]. Other scholars found that mHealth, and other digital health initiatives, represent a “Eurocentric point of view” that excludes “local understanding of needs” [68]. Our findings build upon these conclusions by suggesting another reason these apps are ineffective is that phones and the mobile internet are *not* universally perceived as technologies which support healthcare. Similar to the aforementioned factors underlying mHealth, this sense that mobile devices are technologies that support learning about and managing one’s health, may not apply to users in LMICs. Instead, our findings suggest that in addition to being perceived as sources of fun and entertainment, mobile devices are seen as a financial burden (e.g., constantly having to “feed” them) and a distraction in school. Prior studies of mobile services in Kenya also suggest that phones are primarily perceived as devices that support verbal communication between trusted individuals [81]. Participants’ preference for voice calls is aligned with this observation.

What do these findings mean for HCI/CSCW designers and researchers who perceive mobile phones and other technologies as capable of transforming healthcare? We propose that these communities reevaluate the potential of mHealth apps and consider alternative technologies and design interventions. The disproportionate focus on apps has diverted attention away from other technologies that might benefit from redesign—notably, glucometers. Youth did not seem to need mHealth apps; however, it appeared that access to affordable glucometers would be beneficial. Shifting attention away from mobile phone apps, could also mean allocating resources to create non-digital interventions that do not require a smartphones or the mobile internet, such as paper-based diaries that youth can use to record and track their blood sugar levels and/or T1D educational posters to put in schools. Indeed, efforts to educate the broader public, especially schoolteachers, about T1D seemed more pressing than, for example, sending educational information (via SMS) to youth with the condition. Our findings about the importance of voice communication support the expansion of phone-based programs (e.g., [59]), which allow patients to call in their blood sugar levels to diabetes call centers.

Other significant findings draw attention to the challenges participants encountered when trying to decipher health information on Google and suggest opportunities for researchers to address these immediate and prosaic problems (e.g., designing usable search engine interfaces) instead of longer-term and consequential problems (e.g., improving health outcomes) [76]. Additionally, improving T1D treatment and care should focus on amplifying what seems to already work; that is, providing youth and their caregivers with access to knowledgeable and trusted healthcare providers.

### 5.2 Implications for mHealth Research: Focus on the “Majority World”

We reiterate the need for HCI/CSCW researchers to examine mHealth “from a global perspective,” for reasons not accounted for in prior research [70]. HCI/CSCW design efforts continue to overwhelmingly focus on and benefit “minority world” users; that is, high-income countries typically located in the global North, instead of the “majority world”, or countries in the global South, where 85% of the world’s population lives [46]. Prioritizing design features that primarily align with minority world perspectives reinforces the misconception that these features are the most important (mobile phones are personal devices that are always accessible, etc.). The one device/one owner model did not apply to many of the youth in our study. School bans also meant devices were not always within their reach. The continued focus on design features catering to minority world perspectives may exacerbate health disparities by neglecting the challenges and contexts faced by communities in the majority world. HCI/CSCW researchers must recognize that resources which are in abundant in minority world countries (e.g., smartphones, always-on connectivity, and robust medical systems), are scarce elsewhere.

These and the previously mentioned recommendations run counter to ongoing mHealth and HCI design initiatives, which focus on developing more sophisticated technological solutions for minority world users. These efforts include integrating social media into mHealth apps, syncing these apps with glucometers, as well as recent efforts to investigate how AI and ML can support T1D management [4, 35, 69]. The persistent socioeconomic and infrastructural challenges which clearly affect mobile phone use and access to health information in LMICs can get lost when technological advancement in prioritized in research and design. Instead, prioritizing greater understanding of the everyday challenges T1D youth and their caregivers in LMICs experience, may be key to developing effective health interventions in Kenya and similar contexts.

### 5.3 Limitations and Future Work

This study has limitations. Most participants were affiliated with the Kenya Diabetes Management & Information Centre (DMI). These participants do not represent the broader population of Kenyan youth with T1D, because the vast majority of adolescents in the country remain undiagnosed or receive inadequate treatment [58]. However, we consider this limitation a strength because our findings draw attention to the relative benefits of having access to reliable T1D information and trusted healthcare professionals. Recall bias—that is, participants not accurately remembering past events, should also be considered a limitation.

Further research is needed to explore how undiagnosed and newly diagnosed youth, as well as those who are unaffiliated with DMI, can access the information and services provided to our participants. Future research should also investigate the roles of women, especially mothers and grandmothers, play in managing their children’s T1D. We observed that they bear significant responsibility and are also less likely than men to possess smartphones or the resources to use them. While our study focused on T1D in Kenya, its implications may extend to other diseases in other LMICs. This merits further investigation.

## 6 CONCLUSION

Access to mobile phones around the world has increased dramatically over the last 25 years; this has spurred the development of thousands of mHealth apps, many developed for people with T1D. The purpose of this study was to explore the factors influencing the use—and non-use—of these apps among youth with T1D and their caregivers. We conducted this research in Kenya, an understudied context where the incidence of T1D is rising. Our findings provide additional evidence about the challenges affecting mobile phone use there and in LMICs more broadly. A contribution of this research is that we demonstrate how these challenges conflict with features that are prioritized in mHealth design and which underlie HCI/CSCW design efforts. These findings may undermine the hopes that are invested in mHealth—in particular, whether these technologies can improve health outcomes, especially in LMICs. We observe that HCI and CSCW research has traditionally concentrated on technological advancements within a geographically bounded context. These research communities must evolve if technology is to effectively address pressing global health challenges like T1D.

## CCS Concepts:

• **Human-Centered Computing** → **Human Computer interaction (HCI)** → Empirical studies in HCI

## Footnotes

1 Hyperglycemia is the medical term for high blood sugar levels, typically associated with T1D. Hypoglycemia refers to low blood sugar levels, which can also occur in individuals with T1D and require immediate medical attention.

2 We use the platforms names used in these studies, which were mostly conducted before 2021. However, we acknowledge that Facebook is now referred to as ‘Meta’ and Twitter as ‘X’.

3 The camera was to be used in a later study associated with this project.

4 In Kenya, “data bundles” or “airtime” refer to prepaid packages offered by mobile network operators. These packages provide users with a specific amount of data for accessing the internet on their mobile devices.
